# Study protocol of a randomized controlled clinical trial investigating the effects of omega-3 supplementation on endothelial function, vascular structure, and metabolic parameters in adolescents with type 1 diabetes

**DOI:** 10.1186/s13063-021-05930-1

**Published:** 2021-12-27

**Authors:** Masoud Khorshidi, Aliakbar Sayyari, Beheshteh Olang, Mohammad Reza Alaei, Sakineh Shab-Bidar, Mitra Khalili, Masoud Salehi, Naheed Aryaeian

**Affiliations:** 1 Department of Community Nutrition, School of Public Health, Iran University of Medical Sciences (IUMS), Hemmat Broadway, Tehran, 1449614535 Iran; 2 Pediatric Gastroenterology, Hepatology and Nutrition Research Center, Research Institute for Children’s Health, Shahid Beheshti University of Medical Sciences, Tehran, Iran; 3 Department of Community Medicine, School of Medicine, Shahid Beheshti University of Medical Sciences, Tehran, Iran; 4 Department of Pediatric, School of Medicine, Shahid Beheshti University of Medical Sciences, Tehran, Iran; 5 Department of Community Nutrition, School of Nutritional Sciences and Dietetics, Tehran University of Medical Sciences, Tehran, Iran; 6 Department of Radiology, Shahid Beheshti University of Medical Sciences, Tehran, Iran; 7 Department of Biostatistics, School of Public Health, Iran University of Medical Sciences, Tehran, Iran

**Keywords:** Omega-3, Type 1 diabetes, Endothelial function, Vascular structure, Inflammation, Oxidative stress

## Abstract

**Background:**

Type 1 diabetes is a main health burden with several related comorbidities. It has been shown that endothelial function, vascular structure, and metabolic parameters are considerably disrupted in patients with type 1 diabetes. Omega-3 as an adjuvant therapy may exert profitable effects on type 1 diabetes and its complications by improving inflammation, oxidative stress, immune responses, and metabolic status. Because no randomized clinical trial has examined the effects of omega-3 consumption in children and adolescents with type 1 diabetes; the present study aims to close this gap.

**Methods:**

This investigation is a randomized clinical trial, in which sixty adolescents with type 1 diabetes will be randomly assigned to receive either omega-3 (600 mg/day) or placebo capsules for 12 weeks. Evaluation of anthropometric parameters, flow-mediated dilation (FMD) as an endothelial function marker, carotid intima-media thickness (CIMT) as a vascular structure marker, proteinuria, biochemical factors including glycemic and lipid profile, blood urea nitrogen (BUN), creatinine, high-sensitivity C-reactive protein (hs-CRP), and erythrocyte sedimentation rate (ESR), as well as blood pressure will be done at the baseline and end of the trial. Also, dietary intake and physical activity will be assessed throughout the study. Statistical analysis will be performed using the SPSS software (Version 24), and *P* < 0.05 will be considered statistically meaningful.

**Discussion:**

It is hypothesized that omega-3 supplementation may be beneficial for the management of type 1 diabetes and its complications by reducing inflammation and oxidative stress and also modulating immune responses and glucose and lipid metabolism through various mechanisms. The present study aims to investigate any effect of omega-3 on patients with type 1 diabetes.

**Ethical aspects:**

This trial received approval from Medical Ethics Committee of Iran University of Medical Sciences, Tehran, Iran (IR.IUMS.REC.1400.070).

**Trial registration:**

Iranian Registry of Clinical Trials IRCT20210419051010N1. Registered on 29 April 2021

**Supplementary Information:**

The online version contains supplementary material available at 10.1186/s13063-021-05930-1.

## Background

Type 1 diabetes, a chronic disorder characterized by insulin deficiency, is generally thought to be precipitated by an immune-associated destruction of pancreatic β cells [1]. Genetic, environmental, and immunologic factors are involved in the pathogenesis of type 1 diabetes [2, 3]. In genetically susceptible subjects under the triggering impacts of environmental factors, autoimmune destruction of pancreatic β cells occurs, leading to insulin deficiency and, consequently, overt hyperglycemia. Polyuria, thirst, weight loss, and overt hyperglycemia are the most common symptoms of type 1 diabetes and diagnostic markers in adolescents [4, 5]. Compelling evidence indicates that there is a bidirectional correlation between the severity of type 1 diabetes and the probability of occurrence of its complications, including cardiovascular disorders, nephropathy, and neuropathy [6, 7]. Based on the results of a recent meta-analysis, the prevalence and incidence rate of type 1 diabetes were 9.5% and 15 per 100,000 people worldwide, respectively. The global rise in type 1 diabetes faces several challenges including the provision of insulin in underdeveloped and developing countries [8]. The lifetime economic burden of type 1 diabetes places a high disease cost on healthcare systems. Thus, developing novel therapeutic agents for the management of type 1 diabetes and its associated comorbidities is crucial [9].

Common approaches for the treatment of type 1 diabetes include administration of short, rapid, intermediate, or long-acting insulins, frequent monitoring of blood glucose, regular aerobic exercise, and dietary interventions such as having a healthy-eating plan, carbohydrate counting, and maintaining an ideal weight [10, 11]. Recently, researchers have become interested in exploring novel adjuvant therapies for the management of type 1 diabetes and its complications. Natural compounds possessing antidiabetic properties may be appropriate as adjuncts to existing therapies. Many new dietary supplements such as omega-3 may exert beneficial effects on type 1 diabetes and its related abnormalities, even equal to the effects of commonly known drugs [12]. Omega-3 polyunsaturated fatty acids including eicosapentaenoic acid and docosahexaenoic acid from seafood and alpha-linolenic acid from plant sources have several pharmacological properties, like antioxidant, anti-inflammatory, anti-tumor, anti-depressant, antihypertensive, and lipid-lowering effects [13]. They also enhance various types of metabolic disorders underlying the development of diabetes [14]. An in vivo study performed by Bi et al [15] demonstrated that omega-3 polyunsaturated fatty acids could potentially ameliorate type 1 diabetes and autoimmunity by decreasing serum concentrations of inflammatory biomarkers and modulating the differentiation of T helper and CD4^+^ T cells. Moreover, the results of an observational study by Norris et al. [16] exhibited that dietary intake of omega-3 fatty acids is correlated with declined risk of islet autoimmunity in children at elevated genetic risk of type 1 diabetes.

The present clinical study aims to assess the effects of omega-3 supplementation on endothelial function, vascular structure, and metabolic parameters in adolescents with type 1 diabetes. The primary objective of this research includes the assessment of flow-mediated dilation (FMD) as an endothelial function marker.

## Material and methods

### Study population

Adolescents with type 1 diabetes will be recruited from Mofid Children’s Hospital, Tehran, Iran, by an investigator. The investigator will conduct study assessments and screen study patients. The patients will be diagnosed by a specialist. It should be noted that because of the simultaneous occurrence of the coronavirus disease 2019 (COVID-19) pandemic and implementation of this study, researchers should expand their presence time in Mofid Children’s Hospital for achieving adequate participant enrollment to reach the target sample size. Patients that satisfy the inclusion criteria will be eligible to participate in this research. The inclusion criteria are considered as follows: adolescents aged between 10 to 18 years, whose body mass index (BMI) for age *Z*-score ranges between 5 and 85%, diagnosis of type 1 diabetes by a specialist, receiving insulin therapy, at least 5 years have passed since the diagnosis of type 1 diabetes, and a signed written informed consent form for participation in this trial. The exclusion criteria include subjects who are pregnant or breastfeeding, those affected by endocrine, metabolic, blood clotting, or any acute disorder, and patients who had diabetic ketoacidosis or hypoglycemia (blood sugar level lower than 50 mg/dl) in the last 12 or 3 months, respectively. In addition, subjects who consumed antihypertensive, anti-inflammatory, anticoagulant, lipid-lowering, and weight-lowering agents and antioxidant and omega-3 supplements in the last 6 months prior to the study will be excluded. Patients with fish or seafood allergies and smokers will also be excluded. Participants who are unwilling to continue collaborating on the trial or have poor compliance (less than 80%) with the assigned intervention will be withdrawn from follow-up.

### Study design

**Fig. 1:**
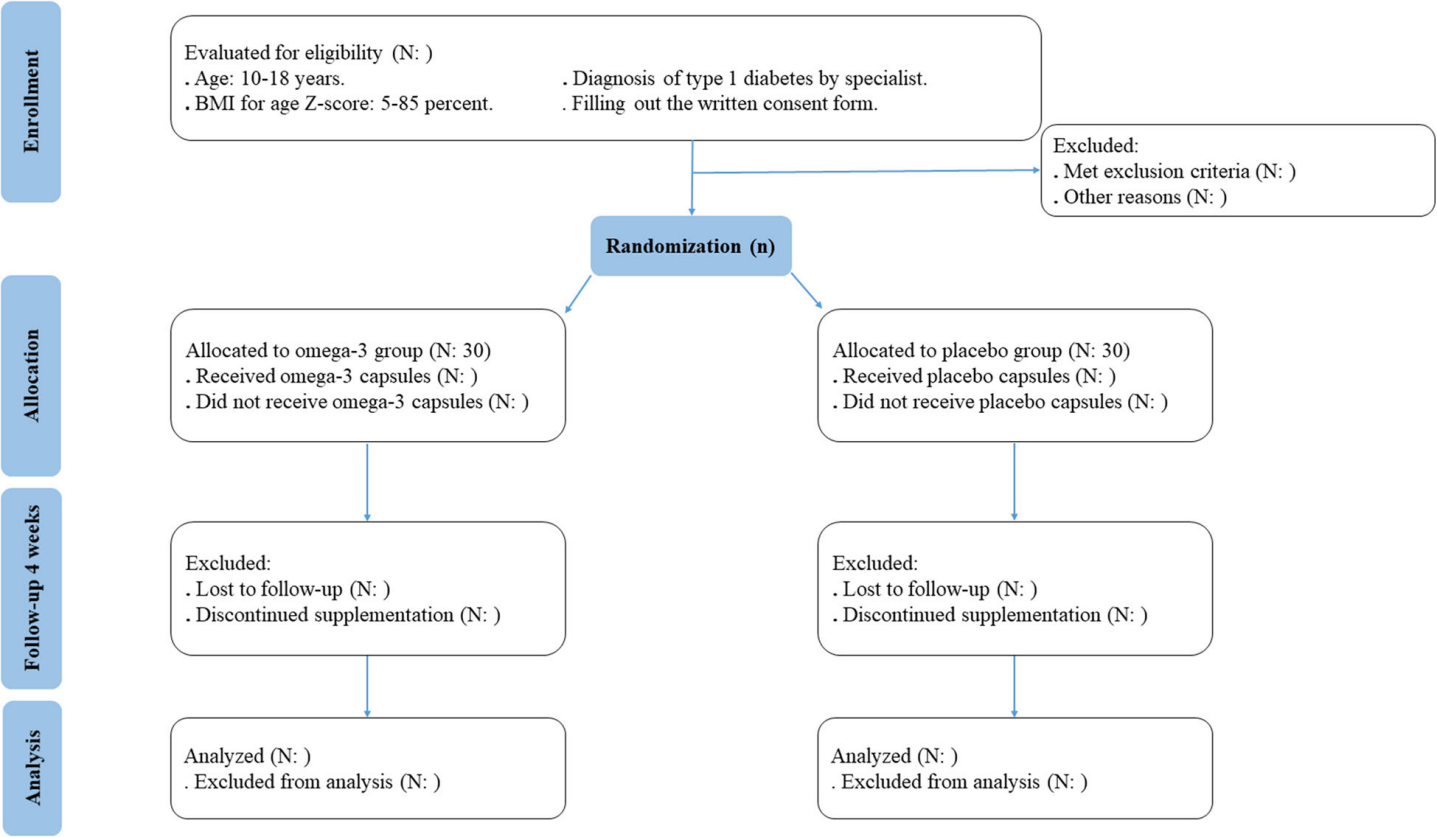
Adapted CONSORT. BMI, body mass index

**Fig. 2:**
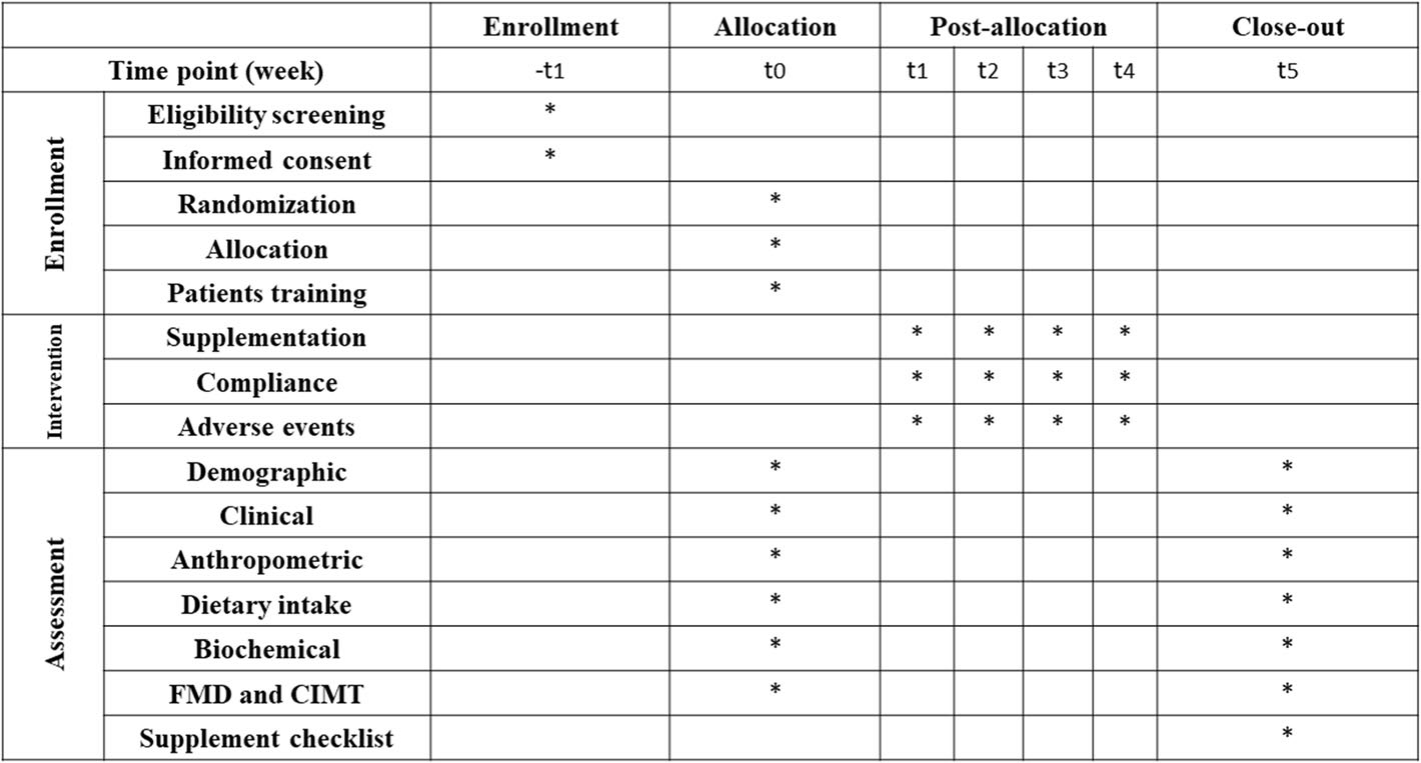
Participant timeline. FMD, flow-mediated dilation; CIMT, carotid intima-media thickness

This is a two-arm, parallel-group, randomized, double-blind, placebo-controlled, clinical trial and will be carried out at Mofid Children’s Hospital, Tehran, Iran. The study protocol has been developed based on Standard Protocol Items: Recommendations for Interventional Trials (SPIRIT) 2013 checklist (supplemental file 1). Study flow chart of enrolment, allocation, intervention, and assessment and participant timeline are presented in Fig. 1 and Fig. 2, respectively. Written informed consent will be obtained from participants or their legal guardians before participating in the research. This investigation was approved by the Medical Ethics Committee of Iran University of Medical Sciences (IR.IUMS.REC.1400.070) and also registered on the Iranian Registry of Clinical Trials website (identifier: IRCT20210419051010N1). In addition, any amendments to the present study protocol which are related to the safety and/or benefit of patients will require confirmation by the aforementioned departments before the trial is conducted. Any alteration in the study protocol will be sent to the Trials journal.

### Randomization and blinding

After obtaining the informed consent and discussing the objectives of the research, eligible subjects (*n* = 60) will be divided into two equal groups, stratified by sex and the amount of daily insulin intake using stratified block randomization, with 1:1 allocation ratio. The block randomization will be conducted by an assistant and the intervention allocation will be blinded for both the investigators and participants. The sequence of the blocks will be prepared for each stratum using a random numbers table. All patients are randomly allocated to an intervention (Omega-3) group or a placebo group. The manufacturer who is responsible for preparing the supplements will be asked to assign a unique code on the cans containing omega-3 or placebo.

### Intervention

Sixty eligible adolescents with type 1 diabetes will be randomly assigned to the omega-3 (*n* = 30) and placebo groups (*n* = 30). Omega-3 capsules (600 mg) and identical placebo capsules will be respectively taken by patients in the intervention and control group once a day, for 12 weeks. The Karen Pharma & Food Supplement company will provide omega-3 capsules. Omega-3 and placebo capsules will be similar in weight, size, shape, taste, color, and odor. Placebo capsules will be also produced by Karen Pharma & Food Supplement company and contain 600 mg oral glycerin. Naringenin and placebo capsules will be given to the study patients for every one month in study visits. There are four study visits in our trial (before the intervention, after 1 month of intervention, after 2 months of intervention, and at the end of the intervention). Study patients will be informed how to use their supplements, and they will be weekly followed by phone calls. Compliance will be evaluated based on unused supplements by each person. The remaining capsules will be counted to determine total supplement intake. Furthermore, patients will be asked about any adverse events experienced following omega-3 consumption. If the reported adverse events are related to omega-3 consumption, participants will be asked to stop taking supplements, and they will be immediately referred to a specialist for therapy. It should be noted that there will be no dietary recommendation or dietary regimen for study patients.

### Sample size calculation

Considering a type I error of 5%, a power of 90%, and the changes in FMD values as one of the primary outcomes [17], the sample size was computed to be 25 for each study group based on the two-sided t-test. To compensate for an approximate attrition rate of 20% throughout the research, we rise the final sample size to 30 adolescents in each group.

$$ n=\frac{{\left({Z}_{1-\alpha /2}+{Z}_{1-\beta}\right)}^2\times \left({S}_1^2+{S}_2^2\right)}{d^2} $$

(*α* = 5%, 1-β = 90%, *SD*_1_ = 2.7, *SD*_2_ = 2.7, *d* = 2.5)

### Outcomes

The primary outcome of the current trial is the alteration in the value of FMD as an endothelial function marker at the end of the trial compared to the baseline value. Alterations in carotid intima-media thickness (CIMT), anthropometric indices including weight, height, and BMI for age *Z*-score, lipid profile including triglyceride (TG), high-density lipoprotein cholesterol (HDL-C), low-density lipoprotein cholesterol (LDL-C), and total cholesterol (TC), glycemic parameters including fasting blood sugar (FBS), hemoglobin A1C (HbA1C), fasting blood insulin (FBI), hemostatic model assessment of insulin resistance (HOMA-IR), and quantitative insulin sensitivity check index (QUICKI), plasma levels of high-sensitivity C-reactive protein (hs-CRP) and erythrocyte sedimentation rate (ESR), blood pressure, BUN, creatinine, proteinuria, energy intake, and consumption of nutrients are considered as secondary outcomes. Furthermore, at the beginning of the research, medical history and demographic factors including age, sex, race, education level, and marital status will be asked from all the patients or their legal guardians.

### Assessment of variables

At the onset and end of the trial, body weight and height will be measured to the nearest 0.1 kg and 0.1 cm using the Seca scale and stadiometer (Seca), respectively. Individuals will be measured barefoot and wearing light clothing. BMI for age *Z*-score is computed by dividing weight in kilograms by height in meters squared according to age in the curve standard using WHO growth standards (www.who.int/childgrowth). A 24-h dietary recall, a valid instrument for the evaluation of diet, will be completed by patients at the beginning and end of intervention in face-to-face interviews and used for dietary assessment. Information about dietary consumption will be analyzed using Nutritionist IV software. The physical activity questionnaire for children (PAQ-C) and physical activity questionnaire for adolescents (PAQ-A) which have been validated in Iran will be applied to estimate participant physical activity levels. The PAQ-C which is appropriate for ages 8–14 provides a physical activity score derived from 9 items, each scored on a 5-point scale. The PAQ-A, which is appropriate for ages 14–20, provides a physical activity score derived from 8 items, each scored on a 5-point scale. Physical activity will be reported in three categories, namely low activity, moderate activity, and high activity levels, based on the PAQ-A scoring protocol [18, 19]. Blood pressure will be measured using a mercury sphygmomanometer after at least 5 min of resting. The measurement will be taken on two occasions, and the mean of the two will be considered as the individual’s blood pressure.

Blood samples (10 mL) will be drawn following 12-h overnight fasting and centrifuge at 3000 rpm for 5 min to extract serum samples pre-and post-intervention. The concentrations of TC, TG, and HDL-C will be evaluated using commercial kits (DIALAB Inc. kit, Vienna, Austria) before and after the intervention. Serum LDL-C values will be calculated using the Friedewald formula. The concentrations of FBI and FBS will be measured using commercial enzyme-linked immunosorbent assay (ELISA) kit (IBT, USA) and glucose oxidase method, respectively. HbA1c will be assessed using commercial kit (BioRex Inc., Tehran, Iran) by auto-analyzer (Mindray auto hematology analyzer). Moreover, BUN and creatinine will be examined by diacetyl monoxime method and enzymatic method, respectively. ESR will be evaluated by Westergren method using a special tube. In addition, serum hs-CRP levels will be assessed using ELISA kit (Paadco Inc. kit, Tehran, Iran) pre-and post-intervention. Also, the following formulas will be used to determine QUICKI and HOMA-IR. Proteinuria will be evaluated by albumin dipstick through collecting urine samples.

QUICKI: 1/(log (fasting insulin μU/ml) + log (fasting glucose mg/dl))

HOMA-IR: [fasting insulin (μU/ml) × fasting glucose (mg/dl)]/405

FMD and CIMT will be assessed by an expert radiologist. For evaluating FMD, after an overnight fasting and 10 min rest, patients will lie down in the supine position in a quiet, temperature-controlled room and put their right hand in a comfortable position on a surface for imaging the brachial artery. Ultrasound imaging of the brachial artery will be done by Doppler ultrasound (Samsung Medison UGEO H60, Seoul, South Korea) and in longitudinal section, 2 cm above of antecubital fossa. An anterior and posterior section will be selected between the lumen and the vessel wall for clear imaging, and after that, the diameter of vessel will be measured from the anterior part to the posterior part between Media and Adventitia. A baseline rest image will be acquired and the arterial diameter will be measured. For the second scan, the cuff of the sphygmomanometer will be closed around the arm and a 5-minute occlusion of at least 50 mm Hg above the systolic pressure will be applied to the vessel. Finally, 90 seconds after the cuff will be opened, the second measurement will be performed. The following formula will be used to calculate FMD, where d1 is the baseline brachial artery diameter and d2 is brachial artery diameter after 90s of cuff release.

FMD: (d2 − d1) × 100/d1

To assess CIMT, the thickness of two inner layers of Intima and Media artery will be measured by Doppler ultrasound (Samsung Medison UGEO H60, Seoul, South Korea). The distance between the main edge of the first line and the main edge of the second line in the carotid artery will be considered as CIMT. To increase the accuracy, all measurements will be repeated for 4 times.

### Data management and monitoring

A clinical trial monitor will occasionally supervise the study progress and ensure that patient rights and well-being are safeguarded. The clinical trial monitor will also supervise that the protocol, ethical requirements, standards, and regulations are being followed, the essential documentation is available, and collected data is accurately recorded. One of the researchers will check the coding, security, and storage of data. In addition, he/she will evaluate data entry and data values twice. If any participant reports the occurrence of adverse events, more information is required to make decision about excluding the participants from the trial. Unblinding is permissible in this situation based on the Medical Ethics Committee criteria [20].

### Statistical analysis

Statistical analysis of all data will be done by SPSS statistical software (SPSS Inc., Chicago, IL, USA, version 24), and *P* values less than 0.05 will be considered statistically significant. The Kolmogorov-Smirnov test will be used for assessing the normality of the data. Quantitative and qualitative variables will be presented as mean (standard deviation) or median (25th–75th percentile) and frequency (percentage), respectively. To examine differences in qualitative variables between omega-3 and placebo groups, we will apply the chi-square test. We will use the independent sample *t*-test or Mann-Whitney *U* test to determine differences in numerical variables between the omega-3 and placebo groups. In addition, aforementioned tests will be used to compare between-group alterations in outcome variables. To do within-group comparison, we will use the paired-sample *t*-test or its nonparametric equivalent, the Wilcoxon test. General linear model will be applied to adjust the effects of confounding factors. To control confounders and strengthen our findings, any alterations in dietary intakes and physical activity levels will be taken into consideration throughout the trial.

## Discussion

The role of omega-3 supplementation in the treatment and management of diabetes is still controversial. A recent preliminary trial investigating the effects of omega-3 in adults with type 1 diabetes demonstrated no significant changes in vascular health, glucose hemostasis, and metabolic parameters. However, limitations of the mentioned study including relatively small sample size, high number of subjects lost to follow-up, and the broad selection of people with different treatment regimens and duration of disease may cause bias in obtained results [21]. Another study performed by Casanova et al. [22] concluded that omega-3 supplementation improved arterial stiffness and endothelial function in hypertensive patients with high cardiovascular risk. Also, endothelial function was improved in endurance-trained athletes by a 3-week omega-3 supplementation [23].

Based on suspected mechanism of action of omega-3 reviewed in previous studies, we hypothesize that omega-3 may improve endothelial function, vascular structure, and metabolic parameters in adolescents with type 1 diabetes by attenuating the inflammatory response through suppression of the nuclear factor-kB (NF-kB) signaling pathway, reduction of oxidative stress via upregulating genes encoding cytoprotective antioxidant proteins such as heme oxygenase 1 (HO-1) and glutathione peroxidase (GPx), modulation of immune responses through decreasing adhesion molecule expression, adhesive interactions between leukocytes and endothelial cells, chemotactic response of leucocytes and T cell reactivity, and regulation of glucose and lipid metabolism by elevating fatty acid oxidation, decreasing the expression of sterol regulatory element binding protein-1c (SREBP-1c) and de novo lipogenesis, and preserving hepatic insulin sensitivity [24–28]. According to aforementioned hypothesize, the present trial is designed. Suggesting a natural compound as an option for the management and treatment of type 1 diabetes is one of the novel characteristics of the current clinical study. In addition, this research may lead to the cost-lowering effects due to the high burden and prevalence of diabetes in Iran and worldwide.

### Strengths and weaknesses

The strengths of the present study include using a randomized, double-blind, placebo-control design and also examining the effects of omega-3 supplementation on endothelial function indices and metabolic parameters in adolescents with type 1 diabetes for the first time. Also, as type 1 diabetes is a major health problem, thus there is a need to explore promising therapeutic agents for the management of the illness and its comorbidities. However, there are some weaknesses in this research. First, despite consideration given to any alteration in dietary intake or physical activity for statistical analysis, participants' self-reporting dietary intake and physical activity may impact the results. Second, no compliance biomarker for omega-3 intake will be measured. Finally, it might be advisable for the duration of omega-3 supplementation to be longer in order to observe an effect on study biomarkers. It is hoped that this trial will provide scientific evidence in support of omega-3 intervention for the management of type 1 diabetes and its consequences in adolescents suffering from the disease.

### Trial status

Recruitment started on 20 May 2021 and is expected to be completed by February 2022.\

## Supplementary Information

**Additional file 1:.** SPIRIT 2013 Checklist: Recommended items to address in a clinical trial protocol and related documents

## Data Availability

Not applicable.

## Abbreviations

FMD: Flow-mediated dilation
CIMT: Carotid intima-media thickness;
BUN: Blood urea nitrogen
hs-CRP: High-sensitivity C-reactive protein
ESR: Erythrocyte sedimentation rate
BMI: Body mass index
TG: Triglyceride
HDL-C: High-density lipoprotein cholesterol
LDL-C: Low-density lipoprotein cholesterol
TC: Total cholesterol
FBS: Fasting blood sugar
HbA1C: Hemoglobin A1C
FBI: Fasting blood insulin
HOMA-IR: Hemostatic model assessment of insulin resistance
QUICKI: Quantitative insulin sensitivity check index
HO-1: Heme oxygenase 1
GPx: Glutathione peroxidase
NF-kB: Nuclear factor-kB
SREBP-1c: Sterol regulatory element binding protein-1c
PAQ-C: Physical activity questionnaire for children
PAQ-A: Physical activity questionnaire for adolescents
ELISA: Enzyme-linked immunosorbent assay
